# High dose versus low dose standardized cranberry proanthocyanidin extract for the prevention of recurrent urinary tract infection in healthy women: a double-blind randomized controlled trial

**DOI:** 10.1186/s12894-021-00811-w

**Published:** 2021-03-23

**Authors:** Asma Babar, Lynne Moore, Vicky Leblanc, Stéphanie Dudonné, Yves Desjardins, Simone Lemieux, Valérie Bochard, Denis Guyonnet, Sylvie Dodin

**Affiliations:** 1 CHU de Québec-Laval University Research Center, Québec City, Canada; 2 Department of Social and Preventive Medicine, Faculty of Medicine, Laval University, Québec City, Canada; 3 Institute of Nutrition and Functional Foods, Laval University, Québec City, Canada; 4 Diana Food, Rennes, France; 5 Diana Nova, Paris, France; 6 Department of Obstetrics and Gynecology, Saint-Francois D’Assise Hospital, Laval University, 10 Espinay Road, Québec City, G1V 0A6 Canada

**Keywords:** Women, Urinary tract infections, Proanthocyanidins, Cranberry, Prevention

## Abstract

**Purpose:**

Our objective was to assess the efficacy of a high dose cranberry proanthocyanidin extract for the prevention of recurrent urinary tract infection.

**Material and methods:**

We recruited 145 healthy, adult women with a history of recurrent urinary tract infection, defined as ≥ 2 in the past 6 months or ≥ 3 in the past 12 months in this randomized, controlled, double-blind clinical trial. Participants were randomized to receive a high dose of standardized, commercially available cranberry proanthocyanidins (2 × 18.5 mg daily, n = 72) or a control low dose (2 × 1 mg daily, n = 73) for a 24-week period. During follow-up, symptomatic women provided urine samples for detection of pyuria and/or bacteriuria and received an appropriate antibiotic prescription. The primary outcome for the trial was the mean number of new symptomatic urinary tract infections during a 24-week intervention period. Secondary outcomes included symptomatic urinary tract infection with pyuria or bacteriuria.

**Results:**

In response to the intervention, a non-significant 24% decrease in the number of symptomatic urinary tract infections was observed between groups (Incidence rate ratio 0.76, 95%CI 0.51–1.11). Post-hoc analyses indicated that among 97 women who experienced less than 5 infections in the year preceding enrolment, the high dose was associated with a significant decrease in the number of symptomatic urinary tract infections reported compared to the low dose (age-adjusted incidence rate ratio 0.57, 95%CI 0.33–0.99). No major side effects were reported.

**Conclusion:**

High dose twice daily proanthocyanidin extract was not associated with a reduction in the number of symptomatic urinary tract infections when compared to a low dose proanthocyanidin extract. Our post-hoc results reveal that this high dose of proanthocyanidins may have a preventive impact on symptomatic urinary tract infection recurrence in women who experienced less than 5 infections per year.

*Trial registration*: Clinicaltrials.gov, identifier NCT02572895

## Introduction

Urinary tract infection (UTI) is one of the main reasons for emergency medical consultation. One in three women over the age of 18 will experience a UTI, and many of them will have repeated infections [1]. Increasing resistance of pathogens to prescribed antibiotics, both in treatment and prophylaxis, as well as the side effects of antibiotics, reinforce the demand for alternatives that are effective and well tolerated [2].

Cranberry products are the most promising natural health alternatives for the prevention of UTIs [3]. Cranberry has been shown to inhibit the adhesion of uropathogenic *Escherichia coli* to uroepithelial cells [4]. In vitro, cranberry proanthocyanidins (PACs) with type A-linkages, have been identified as responsible for this anti-adhesion effect [5]. Clinical trials have been conducted to test the efficacy of cranberry products, mainly in the form of juices, but their results remain discordant [3]. This discrepancy is mainly explained by a lack of compliance, lack of statistical power and variable PAC concentrations in the tested products. Indeed, PAC concentrations are not disclosed in the majority of clinical trials. According to ex vivo clinical studies (dose–effect studies evaluating the optimal dose for urine anti-adhesion effect), the quantification of PACs requires standardized, reproducible methods and should be at least 36 mg/day [5, 6]. We hypothesize that the efficacy of cranberry products on the prevention of recurrent UTIs in women could be improved with the use of an optimal PACs dose (standardized at 2 × 18.5 mg/day).

## Materials and methods

The Cranberry Extract for Prevention of Recurrent UTI Trial (PACCANN) was a randomized, double blind, controlled, clinical trial performed at the Institute of Nutrition and Functional Foods (INAF). The study was conducted according to the guidelines of the Helsinki Declaration (2013 revision). The protocol, consent form and all procedures were approved by the institutional ethics committee of Laval University. Written informed consent was obtained from all study participants. The protocol was registered on ClinicalTrials.gov on October 9, 2015 (NCT02572895) and has been published in BMC Urology [7].

### Study population

We enrolled sexually active non-pregnant women aged 18 years and over presenting with recurrent UTI as diagnosed by a physician (defined as ≥ 2 UTIs in the past 6 months or ≥ 3 UTIs in the past 12 months). Women were recruited in the Laval University community in Quebec City, Canada, through e-mail list serves and local clinician referrals as well as posters in medical clinics, social media, paid advertising and word of mouth. Eligibility of potential participants was verified by the study coordinator according to inclusion and exclusion criteria (Table 1). The risks and benefits of the study have been thoroughly discussed and the consent form was signed at the first of three visits at INAF.

**Table 1:** Admissibility criteria

*Inclusion criteria*
Sexually active healthy women
Aged 18 years and older
Recent history of recurrent urinary tract infections^a^
≥ 2 UTIs in the past 6 months and/or
≥ 3 UTIs in the past 12 months
No consumption of cranberry product, polyphenol or antioxidant supplements in the last 2 weeks
*Exclusion criteria*
Pregnancy
History of anatomical urogenital anomalies, urogenital tract surgery
History of acute or chronic renal failure, nephrolithiasis
History of intestinal diseases causing malabsorption
Anticoagulant medication in the last month
Known allergy or intolerance to cranberry

^a^UTIs diagnosed by a clinician and treated with antibiotic therapy

### Study product

The high dose intervention consisted of twice daily intake of commercially available 120 mg Urophenol™, a purified cranberry extract from whole fruit (*Vaccinium macrocarpon* Aiton) standardized at min 15% PACs. The control dose was standardized at 1% PACs which is comparable to the majority of cranberry products approved by Health Canada [8]. Cranberry PACs were manufactured by Nutra Canada (now part of Diana Food Canada) and similar in size, smell and taste. Capsules were distributed in opaque packaging in order to conceal slight colour variations from the research team. Total PAC content of each treatment was validated at INAF’s analytical laboratory using the 4-dimethylaminocinnamaldehyde (BL-DMAC) method [9] (“Appendix 1”). PACs were also characterized by normal-phase analytical HPLC coupled with fluorescence detection, as previously described [10].

### Randomization

Concealed randomization was generated using computer assisted randomization by blocks of 10. Eligible women were assigned 1:1 to either high PAC (2 × 18.5 mg capsules per day) or low PAC (2 × 1 mg capsules per day) content cranberry capsules for 24 weeks. All clinical investigation, laboratory analysis, data collection and assessment were blinded to the randomization allocation.

### Clinical follow-up

Each visit (0, 12 and 24 weeks) included a short questionnaire documenting socio-demographic characteristics (T = 0 only), medication and natural health product intake, quality of life (SF-12) [11], risk factors for UTIs, and a validated food frequency questionnaire [12] modified for our study to specifically include sixty-one foods containing PACs. Participants were instructed to obtain a midstream urine sample on which dipstick urinalysis and pregnancy tests were performed.

During their participation, women were asked to contact the study coordinator if they presented symptoms of UTI to schedule a visit at INAF in order to confirm the clinical diagnosis, provide a urine sample and receive an appropriate antibiotic prescription. A dipstick urinalysis using Chemstrip 9 (Roche Diagnostics USA) was used to confirm pyuria and urine samples were outsourced to the Laval University Hospital Center microbiology laboratory for culture. In line with the pragmatic aspect of this trial, women who were unable to present themselves to the research facility during a symptomatic episode were provided with an empiric antibiotic by prescription of the clinician.

Women that discontinued the intervention were asked to present themselves at the 12 and 24-week visit to complete intention to treat analysis. All participants were asked not to consume other products containing cranberry derivatives for the duration of the study.

### Outcomes

The primary outcome was the number of symptomatic UTIs during the 24-week follow-up period. Symptomatic UTI was defined as acute urinary symptoms such as urine frequency, urgency, dysuria, pelvic pain, and hematuria in the absence of alternate diagnoses as assessed by study staff. The choice of symptomatic UTI was based on local [13] and international guidelines [14] as well as on realistic clinical settings in North America where empirical therapy is prescribed on the basis of clinical symptoms [14]. This outcome increased our capture of UTI episodes and trial conduct as we anticipated that certain women would be unable to present themselves to the research facilities to provide a urine sample.

Secondary outcomes were symptomatic UTI with pyuria and symptomatic UTI with bacteriuria. Women who presented both symptoms and a positive leukocyte esterase dipstick result, were diagnosed as having symptomatic UTI with pyuria. Episodes were categorized as symptomatic UTI with bacteriuria in the presence of ≥ 10^3^ CFU/ml of uropathogenic bacteria. Women with antibiotic treatment for symptomatic UTI during the study period continued to take the cranberry capsules and remained in the study for 24 weeks.

### Compliance and side effects

Participants completed a daily journal to record compliance and were asked to bring capsule bottles to each visit in order to count remaining capsules. A bi-weekly email reminder was sent to encourage participation. Side effects were evaluated at each visit and participants were asked to document symptoms in their daily journal.

### Sample size and statistical analysis

We estimated that 35% of patients in the control group would present at least one UTI during the 24-week follow-up period [3]. We needed to recruit 126 women to detect a clinically significant difference of 25% between the 2 groups (10% of women assigned to the experimental group would experience at least 1 UTI with a power of 80%). We estimated that 15% of randomized participants would be lost to follow-up [15], therefore 148 women needed to be recruited in order for at least 126 participants to complete the 24-week intervention.

The Poisson regression model was used to compare the incidence of symptomatic UTI during the 24-week follow-up. A Kaplan Meier estimate with a log-rank test was used to compare time to first UTI between the two treatment arms. Intention to treat analyses were performed in all randomly assigned subjects with the observation time censored at the date that the participant abandoned or the date of last contact (either a scheduled study visit or visit for UTI treatment). All statistical analyses were performed using SAS University Edition software (SAS Institute Inc., Cary, NC, USA).

### Post-hoc and sensitivity analysis

In the case of imbalanced groups, we generated relative rate estimates adjusted for potential confounding variables. Univariate regression analyses were performed for relevant variables collected at the baseline visit and data collected from post-hoc questions. Missing data was excluded from analyses for post-hoc questions. Variables with a *p* value < 0.20 in univariate analysis were included in the multivariate regression model. Interactions with the treatment groups were tested in multivariate regression model using a backward elimination method. Interactions with a *p *value < 0.05 are presented in the results by subgroup of the effect-modifying factor.

The incidence of UTI with pyuria or bacteriuria was estimated using a statistical imputation method for missing urine samples with two extreme assumptions: symptomatic UTI episodes without urine samples were classified as (1) no symptomatic UTI with pyuria or bacteriuria; and (2) symptomatic UTI with pyuria or bacteriuria.

## Results

**Fig. 1:**
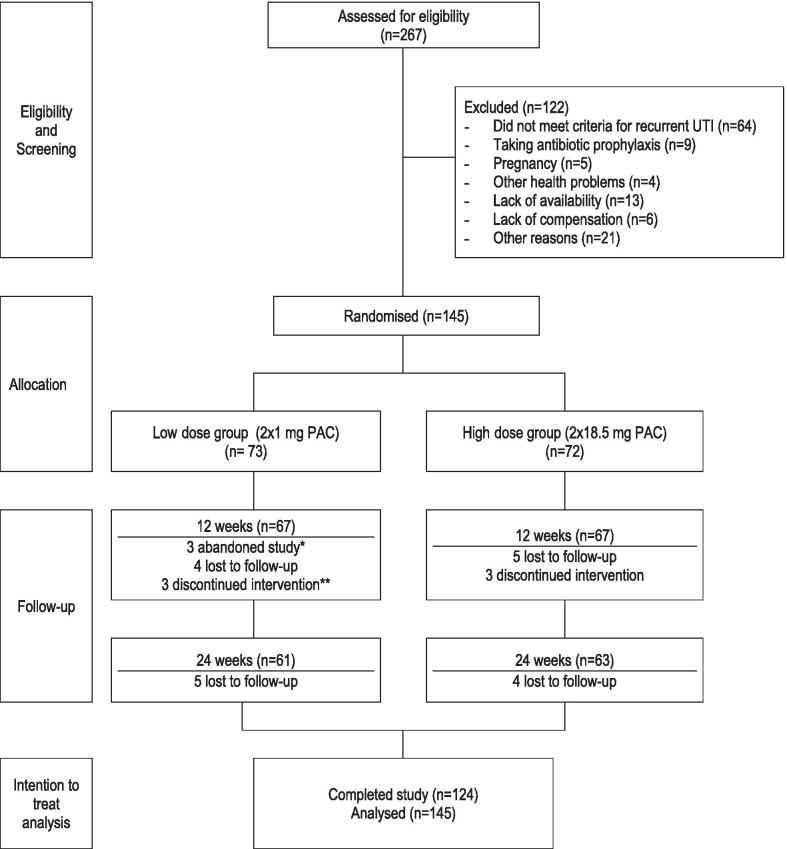
Participant flow diagram. *Women who abandoned the study provided a date and specific reason for their cessation of participation in the trial **Women who ceased the intake of cranberry extract capsules, but presented themselves at study visits

Between August 2015 and December 2016, 267 potential participants were assessed for eligibility, of which 122 were excluded mainly because they did not meet criteria for recurrent UTI (Fig. 1). From August 2015 to April 2017, 145 women were recruited and randomly assigned to consume the high dose or low dose PAC capsules for a 24-week period. Reasons for discontinuing the intervention and abandoning study are shown in Appendices 2 and 3. The groups were well balanced in terms of demographic (Table 2) and clinical (Table 3) characteristics. However, women randomized to the high dose group were significantly younger (mean age 27.2 ± 8.8 years old) than those randomized in the low dose group (mean age 32.5 ± 14.2 years old) (Student t-test, *p* = 0.009).

**Table 2:** Baseline demographic characteristics of participants by study arm

Demographic characteristics	Low dose group, 2 × 1 mg PAC (n = 73)	High dose group, 2 × 18.5 mg PAC (n = 72)	P*
Age (mean ± SD)**,***	32.5 ± 14.2	27.2 ± 8.8	0.009
Age subgroup, years, n (%)			0.028
18–24 years	30 (41.1)	37 (51.4)	
25–44 years	29 (39.7)	31 (43.1)	
> 45 years	14 (19.2)	4 (5.6)	
Age subgroup, years, n (%)			0.003
18–51 years	62 (84.9)	71 (98.6)	
> 51 years	11 (15.1)	1 (1.4)	
Ethnic origin			0.681
Caucasian	65 (89.0)	65 (90.3)	
Non-Caucasian	6 (8.2)	5 (6.9)	
Biracial	2 (2.7)	2 (2.8)	
Marital status			0.230
Single	25 (34.3)	26 (36.1)	
Common law	33 (45.2)	39 (54.2)	
Married	13 (17.8)	7 (9.7)	
Divorced	2 (2.7)	0 (0)	
Has children	21 (28.7)	12 (16.7)	0.082
Living environment			0.998
Urban	50 (68.5)	49 (68.1)	
Suburban	18 (24.7)	18 (25)	
Rural	5 (6.9)	5 (6.9)	
Education			0.814
University	39 (53.4)	42 (58.3)	
College	28 (38.4)	24 (33.3)	
Secondary school	6 (8.2)	6 (8.3)	

*Comparability of numerical and categorical baseline characteristics between groups was assessed with a student’s t-test and ANOVA and chi-squared tests, respectively

**Numbers represent frequency (%) unless otherwise indicated

***Significant difference between groups (student t-test, *p* = 0.009)

**Table 3:** Baseline clinical characteristics of participants by study arm

Clinical characteristics	Low dose group, 2 × 1 mg PAC (n = 73)	High dose group, 2 × 18.5 mg PAC (n = 72)	*P *value*
Number of episodes of UTI in the past 6 months**			0.816
1	13 (17.8)	8 (11.1)	
2	34 (46.6)	37 (51.4)	
3	17 (23.3)	16 (22.2)	
4	6 (8.22)	7 (9.7)	
≥ 5	3 (4.11)	4 (5.6)	
Number of episodes of UTI in the past 12 months			0.249
2	13 (17.8)	7 (9.72)	
3	22 (30.1)	29 (40.3)	
4	11 (15.1)	15 (20.8)	
≥ 5	27 (37.0)	21 (26.17)	
Frequency of sexual intercourse (per week)			0.304
≤ 1	22 (30.1)	14 (19.4)	
2–4	34 (46.6)	41 (56.9)	
5–6	10 (13.7)	13 (18.1)	
≥ 7	7 (9.6)	4 (5.6)	
Number of sexual partners			0.256
0	5 (6.9)	1 (1.4)	
1	67 (91.8)	70 (97.2)	
2	1 (1.4)	1 (1.4)	
Stability in sexual relations			0.987
No stability	3 (4.1)	2 (1.4)	
Stable in the past month	7 (9.6)	6 (8.3)	
Stable in the past 6 months	16 (21.9)	15 (20.8)	
Stable in the past year	46 (63.0)	48 (66.7)	
Prefer not to respond	1 (1.4)	1 (1.4)	
New partner in last month	4 (5.5)	5 (6.9)	0.715
Type of Contraception			0.373
Hormonal contraception	43 (58.9)	53 (73.6)	
Spermicide	1(1.4)	0	
Non hormonal IUD	7 (9.6)	4 (5.6)	
Condom	17 (23.3)	11 (15.3)	
None	5 (6.8)	4 (5.6)	
First UTI before age 15	15 (20.6)	16 (22.2)	0.839
Maternal history of UTI	38 (52.1)	43 (59.7)	0.352
Personal history of recurrent UTI***			0.775
< 1 year	4 (8.2)	2 (4.3)	
1–2 years	9 (18.4)	12 (25.5)	
3–5 years	15 (30.6)	15 (31.9)	
6–10 years	14 (28.6)	10 (21.3)	
> 10 years	7 (14.3)	8 (17.0)	
Missing data	24	25	
Hydration (average litres of water per day)***			0.147
Mean ± SD	1.62 ± 0.75	1.81 ± 0.63	
< 1	9 (15.8)	4 (7.0)	
≥ 1–2	30 (52.6)	27 (47.4)	
> 2–3	13 (22.8)	23 (40.4)	
≥ 3	5 (8.8)	3 (7.0)	
Missing data	16	15	
Alcohol consumption (per week)***			0.140
< 1	7 (12.7)	15 (25.9)	
1–3	28 (50.9)	21 (36.2)	
> 4	20 (36.4)	22 (37.9)	
Missing data	18	14	
Tobacco use***			0.448
Smoker	5 (8.8)	3 (5.2)	
Non-smoker	52 (91.2)	55 (94.8)	
Missing data	16	14	

*Comparability of numerical and categorical baseline characteristics between groups was assessed with a student’s t-test and ANOVA and chi-squared tests, respectively

**Numbers represent frequency (%) unless otherwise indicated

***Factors questioned during the follow-up period with missing data indicated

### Symptomatic UTI

**Fig. 2:**
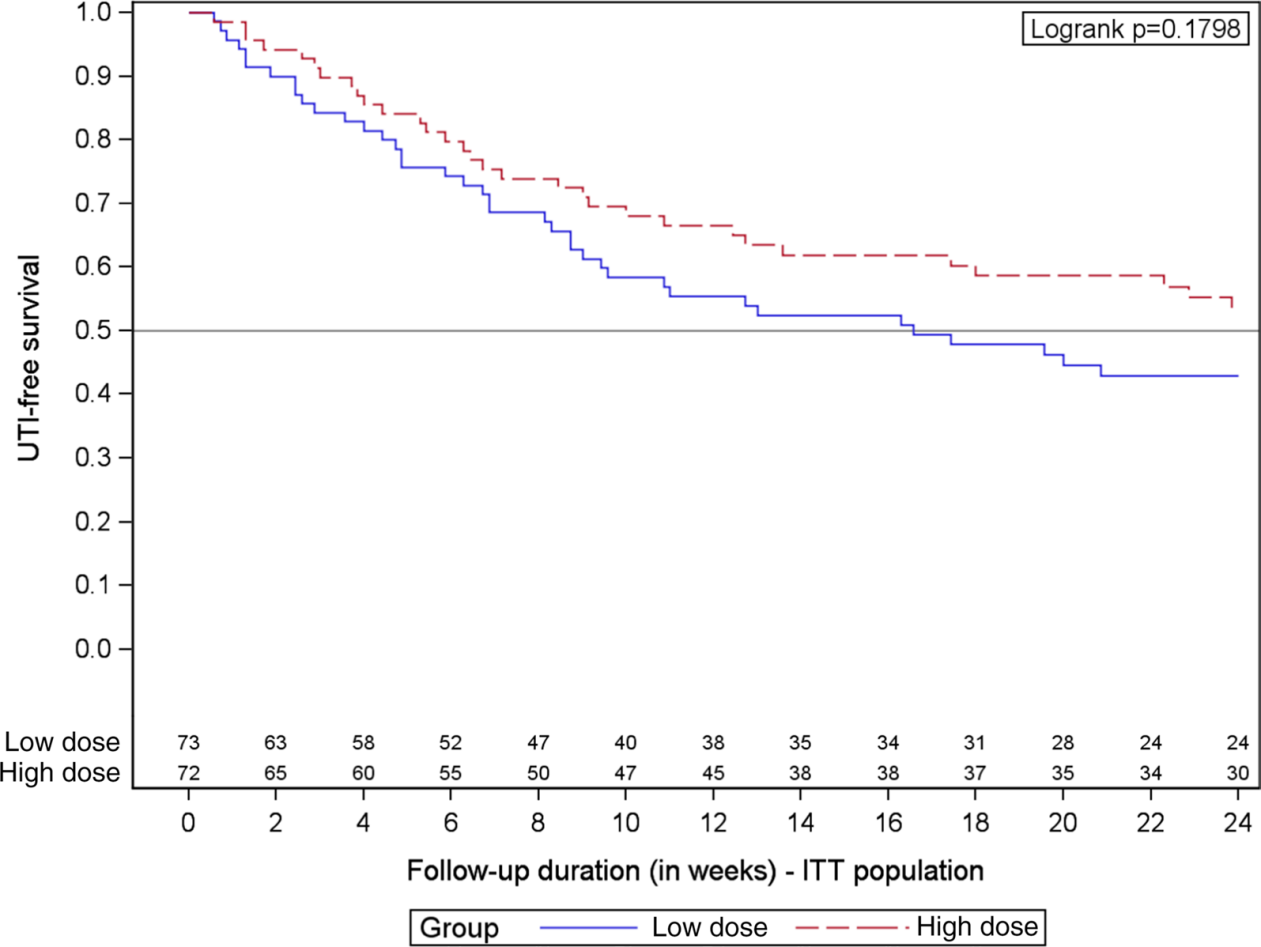
Kaplan Meier analysis of survival to first UTI by treatment group

A total of 45 symptomatic UTIs were diagnosed in the high dose PAC group compared to 59 in the low dose group. The annualized incidence rate of symptomatic UTI for women receiving 2 × 18.5 mg PACs at 24 weeks was 1.48 (95%CI 1.11–1.99) compared to 1.96 (95%CI 1.52–2.53) in women receiving 2 × 1 mg PACs (Incidence rate ratio (IRR) = 0.76, 95% CI 0.51–1.11; Table 4). UTI-free median was 24.0 weeks in the high dose group compared to 16.6 weeks in the low dose group. The hazard ratio for the difference between the number of subjects who had experienced a first symptomatic UTI by the end of the 24-week period was 0.73 (95% CI 0.45–1.16; Fig. 2).

**Table 4:** Incidence of symptomatic UTI at 24 weeks by study arm

	Low dose group, 2 × 1 mg PAC (n = 73)	High dose group, 2 × 18.5 mg PAC (n = 72)	Incidence rate ratio (95% CI)	*p * value
Subjects reporting symptomatic UTI, episodes, n (%)				
0	34 (46.6)	41 (57.0)		
1	26 (35.6)	21 (29.2)		
2	8 (11.0)	7 (9.7)		
3	4 (5.5)	2 (2.8)		
4	0 (0.0)	1 (1.4)		
5	1 (1.4)	0 (0.0)		
≥ 1	39 (53.4)	31 (43.1)		
Total symptomatic UTIs, episodes	59	45		
Follow-up, days (mean ± SD)	151 ± 43	154 ± 39		
Total person-days	10,997	11,088		
UTI, annualized incidence density (95% CI)^a^	1.96 (1.52–2.53)	1.48 (1.11–1.99)	0.76 (0.51–1.11)	0.16
UTI, age-adjusted annualized incidence density (95% CI)^b^	2.28 (1.74–2.98)	1.93 (1.36–2.72)	0.85 (0.57–1.26)	0.42

^a^UTI = urinary tract infection

^b^Incidence rate ratios and *P* values for the number of UTIs per woman-year of observation were determined from the generalized linear model with the Poisson distribution specified

Univariate Poisson regression analysis for total number of symptomatic UTIs and known risk factors are shown in Table 5. After adjustment for age, only the number of UTI in the 12 months preceding enrolment showed a significant interaction between groups. Among participants with less than 5 UTIs in the 12 months preceding enrolment (n = 97), the age-adjusted annualized incidence rate of UTI in the high dose group was 1.32 (95%CI 0.81–2.13) compared to 2.29 (95%CI 1.66–3.16) in the low-dose group (IRR = 0.57, 95%CI 0.33–0.99) (Table 6).

**Table 5:** Univariate poisson regression analysis of total number of symptomatic UTIs and baseline risk factors

Variable	*p* value Incidence rate ratio (95%CI)
Age (ref. = 18–45 years)	*p* = 0.01
45 years and older	1.97 (1.25–3.11)
Number infections past 12 months (ref. < 5)	*p* = 0.02
≥ 5	1.62 (1.1–2.39)
Frequency of sexual intercourse (per week) (ref. < 1)	*p* = 0.12
2–4	0.63 (0.4–0.97)
≥ 5–6	0.78 (0.46–1.32)
Hormonal contraception (ref. = no)	*p* = 0.84
Yes	0.96 (0.65–1.41)
Hydratation (average number of litres of liquid per day) (ref. < 1 L/day)	*p* = 0.19
1–2 L/day	0.57 (0.32–1.02)
≥ 2 L/day	0.66 (0.36–1.18)

**Table 6:** Poisson regression sensitivity analysis of symptomatic UTIs by number of UTI prior to enrolment

	Low dose group, 2 × 1 mg PAC	High dose group, 2 × 18.5 mg PAC	Incidence rate ratio (95% CI) *p *value
Participants with less than 5 UTIs in the 12 months preceding enrolment	n = 46	n = 51	
Number of symptomatic UTI	39	21	
Symptomatic UTI, age-adjusted incidence density (95% CI)^a^	2.29 (1.66–3.16)	1.32 (0.81–2.13)	0.57 (0.33–0.99) *p* = 0.048
Participants with 5 UTIs or more in the 12 months preceding enrolment	n = 27	n = 21	
Number of symptomatic UTI	20	24	
Symptomatic UTI, age-adjusted incidence density (95% CI)^a^	2.23 (1.37–3.61)	3.34 (1.96–5.68)	1.50 (0.81–2.76) *p* = 0.195

^a^Incidence rate ratios and *p* value for symptomatic UTIs per woman-year of observation by number of UTI in the 12 months prior to enrolment (less than 5 UTIs vs greater than 5 UTIs) and adjusted for age group (< 45 years vs ≥ 45 year)

### Symptomatic UTI with pyuria

Data were obtained from women who presented themselves to the research facility in order to provide a urine sample in 70 out of 104 symptomatic UTI episodes. Eighty-one percent of the 70 urine samples obtained presented pyuria as measured by a positive leucocyte esterase dipstick test. No statistically significant reductions in the incidence rate of symptomatic UTI with pyuria were found between treatment groups. In women with less than 5 UTIs in the 12 months prior to enrolment, the daily intake of 2 × 18.5 mg PAC, compared to 2 × 1 mg PAC, was associated with a statistically significant 47% reduction in the age-adjusted incidence rate of symptomatic UTI with pyuria (IRR = 0.54, 95%CI 0.30–0.99), where symptomatic UTI without urine samples were classified as symptomatic UTI with pyuria (Table 7)).

**Table 7:** Incidence of symptomatic UTI with pyuria at 24 weeks by study arm

	Low dose group, 2 × 1 mg PAC	High dose group, 2 × 18.5 mg PAC	Incidence rate ratio (95% CI) *p* value
Symptomatic UTI with pyuria, episodes, n (%)^a^	n = 73	n = 72	
0	50 (68.5)	53 (73.6)	
1	17 (23.3)	14 (19.4)	
2	5 (6.8)	4 (5.6)	
3	0 (0.0)	0 (0.0)	
4	1 (1.4))	1 (1.4)	
≥ 1	23 (31.5)	19 (26.4)	
Total symptomatic UTIs with pyuria, episodes	31	26	
Total person-days	10,997	11,088	
Symptomatic UTI with pyuria, annualized incidence density (95% CI)	1.03 (0.72–1.46)	0.86 (0.58–1.26)	0.83 (0.49–1.40) *p* = 0.49
Symptomatic UTI with pyuria, age adjusted annualized incidence density (95% CI)	1.24 (0.86–1.77)	1.21 (0.77–1.89)	0.98 (0.57–1.68) *p* = 0.94
Subgroup analyses In women with < 5 UTI in past 12 months	n = 46	n = 51	
Symptomatic UTI with pyuria	21	12	
Symptomatic UTI with pyuria, annualized incidence density (95% CI)	1.05 (0.60–1.50)	0.55 (0.24–0.87)	0.53 (0.26–1.07) *p* = 0.08
Symptomatic UTI with pyuria, age-adjusted annualized incidence density (95% CI)	1.27 (0.82–1.95)	0.83 (0.44–1.54)	0.65 (0.31–1.37) *p* = 0.26
Symptomatic UTI with pyuria, episodes, n (%)^b^	n = 73	n = 72	
0	39 (53.4)	45 (62.5)	
1	23 (31.5)	18 (25.0)	
2	6 (8.2)	7 (9.7)	
3	4 (5.5)	1 (1.4)	
4	0 (0.0)	1 (1.4)	
5	1 (1.4)	0 (0.0)	
≥ 1	34 (46.6)	27 (37.5)	
Total symptomatic UTIs with pyuria, episodes	52	39	
Total person-days	10,997	11,088	
Symptomatic UTI with pyuria, annualized incidence density (95% CI)	1.73 (1.32–2.27)	1.28 (0.94–1.72)	0.74 (0.49–1.13) *p* = 0.16
Symptomatic UTI with pyuria, age-adjusted annualized incidence density (95% CI)	2.05 (1.54–2.71)	1.75 (1.22–2.52)	0.86 (0.56–1.32) *p* = 0.48
Subgroup analyses In women with < 5 UTI in past 12 months	n = 46	n = 51	
Symptomatic UTI with pyuria	35	17	
Symptomatic UTI with pyuria, age-adjusted annualized incidence density (95% CI)	1.75 (1.26–2.20)	0.78 (0.41–1.16)	0.45 (0.25–0.80) *p* = 0.01
Symptomatic UTI with pyuria, age-adjusted annualized incidence density (95% CI)	2.1 (1.50–2.94)	1.14 (0.68–1.92)	0.54 (0.30–0.99) *p* = 0.047

^a^Symptomatic UTI episodes without urine sample were considered as no symptomatic UTI with pyuria

^b^Symptomatic UTI episodes without urine sample were considered as symptomatic UTI with pyuria

### Symptomatic UTI with bacteriuria

Urine culture was performed in 61 of the 70 urine samples collected during symptomatic UTI. A positive culture was confirmed in 49% of the 61 urine samples analyzed during symptomatic UTI. No statistically significant reductions in the age-adjusted incidence rate of symptomatic UTI with bacteriuria were found between groups nor in sub-group analyses in women with less than 5 UTIs in the 12 months prior to enrolment (Table 8).

**Table 8:** Incidence of symptomatic UTI with bacteriuria at 24 weeks by study arm

	Low dose group, 2 × 1 mg PAC	High dose group, 2 × 18.5 mg PAC	Incidence rate ratio (95% CI) *p* value
Symptomatic UTI with bacteriuria, episodes, n (%)^a^	n = 73	n = 72	
0	55 (75.3)	63 (87.5)	
1	17 (23.3)	7 (9.7)	
2	1 (1.4)	2 (2.8)	
≥ 1	18 (24.7)	9 (12.5)	
Total symptomatic UTIs with bacteriuria, episodes	19	11	
Total person-days	10,997	11,088	
Symptomatic UTI with bacteriuria, annualized incidence density (95% CI)	0.63 (0.40–0.99)	0.36 (0.20–065)	0.57 (0.27–1.21) *p* = 0.14
Symptomatic UTI with bacteriuria, age-adjusted annualized incidence density (95% CI)	0.76 (0.48–1.21)	0.53 (0.27–1.02)	0.69 (0.32–1.49) *p* = 0.34
Subgroup analyses In women with < 5 UTI in past 12 months	n = 46	n = 51	
Symptomatic UTI with bacteriuria	14	5	
Symptomatic UTI with bacteriuria, annualized incidence density (95% CI)	0.70 (0.42–1.18)	0.23 (0.10–0.55)	0.33 (0.12–0.91) *p* = 0.03
Symptomatic UTI with bacteriuria, age-adjusted annualized incidence density (95% CI)	0.85 (0.50–1.43)	0.35 (0.14–0.89)	0.41 (0.14–1.19) *p* = 0.10
Subjects reporting symptomatic UTI with bacteriuria, episodes, n %)^b^			
0	42 (57.5)	51 (70.8)	
1	22 (30.1)	15 (20.8)	
2	6 (8.2)	4 (5.6)	
3	3 (4.1)	1 (1.4)	
4	0 (0.0)	1 (1.4)	
≥ 1	31 (42.5)	21 (29.2)	
Total symptomatic UTIs with bacteriuria, episodes	43	30	
Total person-days	10,997	11,088	
Symptomatic UTI with bacteriuria, annualized incidence density (95% CI)	1.43 (1.06–1.93)	0.99 (0.69–1.41)	0.69 (0.43–1.10) *p* = 0.12
Symptomatic UTI with bacteriuria, age-adjusted annualized incidence density (95% CI)	1.71 (1.26–2.33)	1.40 [0.93–2.10)	0.81 (0.50–1.32) *p* = 0.40
Subgroup analyses In women with < 5 UTI in past 12 months	n = 46	n = 51	
Symptomatic UTI with bacteriuria	28	13	
Symptomatic UTI with bacteriuria, annualized incidence density (95% CI)	1.40 (0.97–2.03)	0.60 (0.35–1.03)	0.43 (0.22–0.83) *p* = 0.01
Symptomatic UTI with bacteriuria, age-adjusted annualized incidence density (95% CI)	1.70 (1.17–2.46)	0.93 (0.52–1.66)	0.55 (0.28–1.08) *p* = 0.08

^a^Symptomatic UTI episodes without urine sample were considered as no symptomatic UTI with uropathogenic bacteriuria > 10^3^ CFU/ml

^b^Symptomatic UTI episodes without urine sample were considered as symptomatic UTI with uropathogenic bacteriuria > 10^3^ CFU/ml

### Compliance, side effects and adherence to double-blind procedures

Compliance based on number of returned capsules at 24 weeks was similar in both groups (92.9% in the high dose group vs 92.7% in the low dose group, student t-test *p* = 0.9). Compliance according to daily intake journals was comparable in both groups, 87.3% in the high dose group and 88.8% in the low dose group (Student t-test, *p* = 0.6). No serious adverse events occurred in either of the study groups. The only reported side effect, dyspepsia, led to a discontinuation of the intervention of one participant in each group. Participants were asked if they were aware of which treatment group they were assigned to in order to validate the effectiveness of blinding procedures. The majority of women in both groups responded that they were unaware of group allocation as show in “Appendix 4”.

## Discussion

This randomized clinical trial is the first to evaluate the efficacy of a standardized split daily dose of 37 mg cranberry PACs in capsule form on symptomatic UTI. It is of particular interest that this dose was compared to another cranberry extract containing a low concentration of PACs, as often found on the Canadian supplement market to prevent UTI. Our results indicate that the intake of 2 × 18.5 mg PACs daily was associated with a non-statistically significant 24% reduction in the risk of symptomatic UTI compared to a daily dose of 2 × 1 mg PACs during a 24-week follow-up period, similar to the results of a recent meta-analysis [16].

The present study is distinct given that we recruited according to a guideline-based definition of recurrent UTI [17]. Our cohort had a higher mean incidence of UTI prior to study enrolment (mean UTI = 2.4/6 months and 3.9/1 year) compared to similar trials such as Maki et al. [18] (1.65/6 months), Barbosa-Cesnik et al. [19] (1.13 /1 year) or Stothers et al. [20] (2.8/1 year). The proportion of women in the 2 mg PAC group that experienced more than 1 UTI (53.4%) was also greater than the estimated 35% [21] used to calculate our sample size. Considering these key differences with previous reports, we explored if UTI burden at baseline could impact the treatment effect. Statistical analysis confirmed that number of recurrent UTI was indeed a modifying factor. In women who experienced fewer than 5 UTI (mean number = 3.1) in the year prior to enrolment, symptomatic UTIs were significantly reduced by 43%. No significant effect was observed in women with higher past UTI burden.

In women experiencing higher level of UTI (> 5), certain factors that were not measured in our study could explain the mitigated response to PAC intake. It is possible that in these women, UTI recurrence may be the result of complex interactions between bacterial urovirulence and a particular host susceptibility (altered gut microbiota, less efficient adaptive immune response) [22–24]. Considering that PACs are poorly absorbed and are difficult to quantify in the urine, future studies should focus on the effect of these molecules on the gut microbiota, a natural reservoir for uropathogenic *Escherichia coli* [22, 25]. Gut microbiota composition and function largely vary between individuals which might explain differences in individual’s susceptibility to UTI. This could be mediated by a direct effect (e.g. growth inhibition of pathobiont [26], encroachment of uropathogenic bacteria in the gut) or an indirect effect (microbial metabolism of PAC or potential bioactive urinary metabolites). In order to provide mechanistic insight explaining different levels of recurrence, feces and urine samples are being analysed and results will be published in a future article.

Our study was based on medical practice in Quebec whereby many women receive antibiotics without delaying treatment for several days while awaiting urine culture results. Similarly, Maki et al. [18]. compared daily intake of 41 mg PACs from cranberry juice cocktail compared to a placebo beverage on the incidence of symptomatic UTI during a 24-week follow-up. In our study, pyuria was present in 81% and bacteriuria was confirmed in 49% of the urine samples provided by participants during symptomatic UTI. This corresponds to the similar proportion of 80% of symptomatic UTI with pyuria and 60% with bacteriuria in a total of 106 symptomatic UTI episodes presented by Maki et al. [18].

This study had limitations that could influence our findings. The use of a control dose instead of placebo  may explain why we were unable to find a significant reduction in the recurrence of UTI in our cohort with a high UTI burden. A study conducted by Vostavola et al. has shown that 2.8 mg PAC daily can significantly impact the incidence of recurrent UTIs compared to placebo [27]. Moreover, urine cultures were incomplete for a proportion of urine samples provided by symptomatic participants. Urine culture contaminated by improper clean-catch urine technique were excluded from analyses in order to mitigate a risk of detection bias. We also experienced technical issues such as delays in delivery to the microbiology laboratory and improperly stored samples.

## Conclusions

The intake of 2 × 18.5 mg PACs daily was associated with a non-statistically significant 24% reduction in the risk of symptomatic UTI compared to a daily dose of 2 × 1 mg PACs during a 24-week follow-up period. In a subset of participating women with a history of less than 5 UTIs per year, the daily consumption of 2 × 18.5 mg PACs resulted in a significant reduction in the rate of symptomatic UTI during the trial period compared to 2 × 1 mg PAC. These findings need to be tested in women with moderate burden of recurrent UTI who may benefit from a preventive treatment with a split dose of 37 mg/day of PACs from cranberry extract, with few associated side effects. Further investigations are also needed to examine dose-dependent impacts of cranberry PACs for the prevention of recurrent UTI and their effects on the microbiota.

## Data Availability

All data sets will be password protected and only available to project investigators. Data sets, cleaned and blinded of any identifying participant information, as well as the full protocol, will be available after the completion of the trial on request to the contacting author. Data was entered electronically and original study forms will be kept locked at the study site and maintained in storage for a period of 25 years after the completion of the study.

## Appendix 1

See Table 9.

**Table 9:** Cranberry extract composition in polyphenols

Compound (mg/capsule)	Low dose group	High dose group
PAC^a^	1.00	18.50
PAC oligomers^b^	0.35	4.15
PAC polymers^b^	0.55	6.50
Phenolic acids^c^	0.28	2.30
Anthocyanins^c^	0.11	0.55
Flavonols^c^	0.32	1.65

^a^BL-DMAC equivalent A2

^b^HPLC normal phase fluorescence

^c^HPLC inverse phase

## Appendix 2

See Table 10.

**Table 10:** Reasons for abandoning study

Reason	Low dose group (n = 12)
Moved out of province	1
Too difficult to adhere to capsule intake	1
Family disapproval of study participation	1
No explanation given	9
Reason	High dose group (n = 9)
Too difficult to adhere to capsule intake	3
Health problems not related to capsule intake	2
No explanation given	4

## Appendix 3

See Table 11.

**Table 11:** Reasons for discontinuing intervention

Reason	Low dose group (n = 3)
Too difficult to adhere to capsule intake	1
Dyspepsia	1
No reason	1
Reason	High dose group (n = 3)
No reason	2
Dyspepsia	1

## Appendix 4

See Table 12.

**Table 12:** Adherence to double blind procedures

Perception	Low dose group (n = 73)	High dose group (n = 72)
Intervention dose	21 (29)	17 (24)
Control dose	15 (21)	11 (15)
Don’t know	22 (30)	32 (44)
Missing data	15 (21)	12 (17)

Numbers represent frequency (%)
